# STAT3 blockade enhances the efficacy of conventional chemotherapeutic agents by eradicating head neck stemloid cancer cell

**DOI:** 10.18632/oncotarget.5986

**Published:** 2015-11-09

**Authors:** Lin-Lin Bu, Zhi-Li Zhao, Jian-Feng Liu, Si-Rui Ma, Cong-Fa Huang, Bing Liu, Wen-Feng Zhang, Zhi-Jun Sun

**Affiliations:** ^1^ The State Key Laboratory Breeding Base of Basic Science of Stomatology & Key Laboratory of Oral Biomedicine Ministry of Education, Wuhan, China; ^2^ Department of Oral Maxillofacial-Head Neck Oncology, School and Hospital of Stomatology, Wuhan University, Wuhan, China

**Keywords:** STAT3, head neck squamous cell carcinoma, cancer stem cell, S3I-201, chemotherapy

## Abstract

Signaling transducer and activator 3 (STAT3) and cancer stem cells (CSCs) have garnered huge attention as a therapeutic focus, based on evidence that they may represent an etiologic root of tumor initiation and radio-chemoresistance. Here, we investigated the high phosphorylation status of STAT3 (p-STAT3) and its correlation with self-renewal markers in head neck squamous cell carcinoma (HNSCC). Over-expression of p-STAT3 was found to have increased in post chemotherapy HNSCC tissue. We showed that blockade of p-STAT3 eliminated both bulk tumor and side population (SP) cells with characteristics of CSCs *in vitro*. Inhibition of p-STAT3 using small molecule S3I-201 significantly delayed tumorigenesis of spontaneous HNSCC in mice. Combining blockade of p-STAT3 with cytotoxic drugs cisplatin, docetaxel, 5-fluorouracil (TPF) enhanced the antitumor effect *in vitro* and *in vivo* with decreased tumor sphere formation and SP cells. Taken together, our results advocate blockade of p-STAT3 in combination with conventional chemotherapeutic drugs enhance efficacy by improving CSCs eradication in HNSCC.

## INTRODUCTION

Head neck squamous cell carcinoma (HNSCC), which has more than 600,000 newly diagnosed cases per year and a high mortality rate, is the sixth most common cancer worldwide [1]. Despite significant advances in therapeutic approaches including reconstructive surgery, minimally invasive surgery, precisely targeted radiotherapy, chemotherapy, and monoclonal antibody therapy that have been achieved in the last three decades, little improvement has been achieved in the overall survival rates for HNSCC patients [2]. The mortality of HNSCC is mainly caused by the emergence of therapy-resistant local recurrence and local metastasis to cervical lymph node, and occasionally by metastasis in distant organs [2]. Thus, an urgent better understanding of HNSCC tumorigenesis and more efficacy therapeutic target (oncotarget) are needed for improving clinical outcome of this fatal disease.

Accumulating evidence reveals that many types of tumors including HNSCC are frequently composed of heterogeneous cell types and that tumor initiation, growth, metastasis, chemoresistance, and recurrence after therapy are driven by a subpopulation of cells, termed cancer stem cells (CSC) or tumor-initiating cells [3]. CSCs share certain properties with normal stem and/or progenitor cells, while it have accumulated oncogenic mutations and lost normal constraints on growth control. Recent studies have suggested that CSCs play a pivotal role in the development and progression of HNSCC [4]. Accumulated evidence indicated that CSCs, which have not been completely destroyed in conventional chemotherapy, can cause relapse of cancer and regrowth of the tumor [5]. Therefore, the improvement of therapies targeting CSCs may raise hope for the treatment of HNSCC patient. All told, it is essential to develop the corresponding treatment targeting CSCs to enhance the selectivity and efficiency of radiotherapy and chemotherapy. Recent study reported the existence of the side population (SP) cells, which are often used for the identification and isolation of cancer stem-like cells [6]. In addition, previous study identified CD44^+^ [7] and ALDH1^+^ [8] cell population as possible molecular biomarkers of cancer stemloid cells in HNSCC patient.

As a point of convergence for many oncogenic signaling pathways, STAT3 is persistently activated in HNSCC by abnormal signaling of various growth factor receptors [9]. Phosphorylated STAT3 monomers dimerize and translocate to the nucleus [10] to induce transcription of genes involving in cell survival, proliferation, angiogenesis and metastasis in HNSCC, and STAT3 has been proposed as a therapeutic oncotarget [11]. In addition, STAT3 has been validated to affect cancer cell sensitivity to conventional chemotherapeutic agents such as cisplatin (CDDP), [12], paclitaxel [13], imatinib [14], gefitinib [15] and erlotinib [16]. However, the effect of STAT3 inhibition alone or in combination with conventional chemotherapeutic agents on drug-resistant CSCs has still not been well investigated in HNSCC.

In this study, correlation between p-STAT3 and self-renewal markers was explored in human HNSCC. The efficacies of selective STAT3 inhibitor in chemotherapy-enriched HNSCC cancer stemloid cell population were explored *in vitro* and *in vivo*.

## RESULTS

### Activation of STAT3 in human HNSCC is associated with cancer stem cells

**Figure 1:**
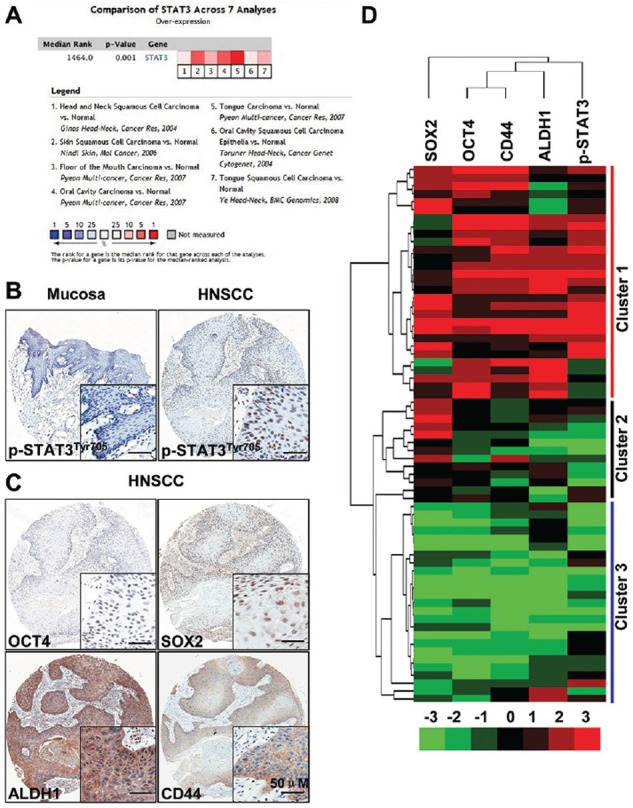
STAT3 signaling is activated in head and neck cancer **A.** Meta-analysis of recent gene expression profiling for STAT3 analyzed by Oncomine where the colored squares indicated the median rank for STAT3 across each analysis in various human cancer. *P* = 0.001. **B.** Over-expression of p-STAT3^Tyr705^ in head neck squamous cell carcinoma (HNSCC, *n* = 43, right) as compared with normal oral mucosa (Mucosa, *n* = 16 left); scale bars, 50 μm. **C.** Representative immunohistochemical staining (IHC) of CD44, ALDH1, OCT4 and SOX2 in HNSCC; Scale bars, 50 μm. **D.** Hierarchical clustering of p-STAT3^Tyr705^, OCT4, SOX2, CD44 and ALDH1. Immunohistochemical staining was clustered with Cluster and Java Treeview.

Previous reports have shown that activation of STAT3 signaling was due to gene mutation and high level phosphorylation were widely expressed in HNSCC [17, 18], while the exact role of STAT3 in HNSCC CSCs is unclear. To determine whether STAT3 pathway expression was associated with human HNSCC, we interrogated the Oncomine database [19] to explore the gene expression of *STAT3* in head neck cancer. Strikingly, the high alternation expression *STAT3* is significantly increased in 17/18 HNSCC datasets (Supplementary Figure S1A). Meta-analysis suggest significant increase of *STAT3* using 7 dataset (*P* = 0.001, Figure 1A). Data retrieved from Tissue Cancer Genome Atlas head neck cancer dataset [20] suggest DNA copy number of *STAT3* significant increase in human HNSCC as compared with control counterpart (*P* = 7.69E-4, Supplementary Figure S1B). Dataset from another 3 independent datasets confirms mRNA level of different location of head neck cancer is significantly higher as compared with oral mucosa (Supplementary Figures S1C–S1E). We started to examine the phosphorylation Status of STAT3 in tyrosine 705 residue. As expected, p-STAT3 was highly expressed in HNSCC (*n* = 43) as compared with normal oral mucosa samples (*n* = 16, *P* < 0.001, Figure 1B and Supplementary Figure S2A) and there was significantly increased in high grade HNSCC (Grade III verse Grade I, *P* < 0.05, Supplementary Figure S2B) as well as in node positive original HNSCC (N1+N2 verse N0, *P* < 0.05, Supplementary Figure S2C), while there was no significant difference between Grade III and Grade II, and no significant difference between Grade II and Grade I. We further investigated the correlation of p-STAT3 with CSCs markers based on previous reports that STAT3 plays crucial roles in the regulation of cancer stem cells. We examined the expression of CSCs self-renewal related markers ALDH1, CD44, OCT4 and SOX2. Interestingly, all these self-renewal markers showed high expression levels in HNSCC tissue as compared with normal mucosa (Figure 1C). The expression of p-STAT3 significantly correlated with CSCs markers OCT4 (*r* = 0.4209, Supplementary Figure S2D), SOX2 (*r* = 0.4310, Supplementary Figure S2E), ALDH1 (*r* = 0.3396, Supplementary Figure S2F), and CD44 (*r* = 0.3961, Supplementary Figure S2G). Besides, to better visualize the correlation of p-STAT3 and CSCs markers, we conducted hierarchical cluster analysis (Figure 1D). Together, these results suggest over-expression of p-STAT3 and the close correlation between p-STAT3 with CSCs self-renewal markers were universal phenomenon in HNSCC, which indicates that p-STAT3 has potential roles in CSCs regulation.

### Blockade of p-STAT3 attenuates cell viability and CSCs phenotype of HNSCC *in vitro*

**Figure 2:**
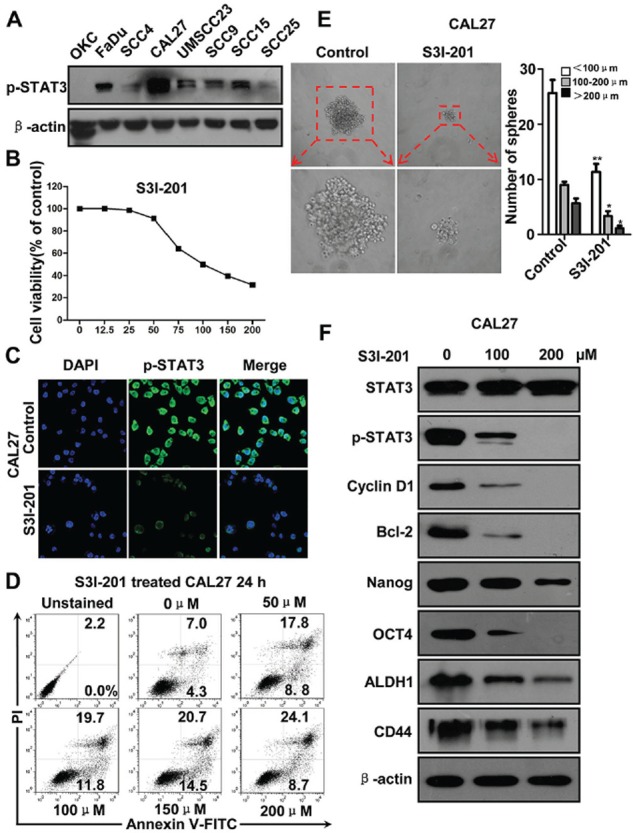
STAT3 inhibition by S3I-201 in HNSCC CAL27 cell line **A.** Western blotting of p-STAT3^Tyr705^ of HNSCC cell lines as compared with oral keratinocyte cell line (OKC). **B.** Cell growth of CAL27 was measured using a CCK8 assay after with S3I-201 for 24 h in different concentrations. **C.** Immunoflurosece shows S3I-201 reduce nuclear expression of p-STAT3^Tyr705^ by confocal microscope. **D.** Representative flow cytometry shows S3I-201 increase apoptosis cells in CAL27 cell line in a dose dependent manner. **E.** Sphere formation analysis shows S3I-201 decrease CAL27 tumor-sphere formation. Mean ± SEM *,*P* < 0.05; **,*P* < 0.01. **F.** Western blotting shows S3I-201 treatment decrease Cyclin D1, Bcl2 and self-renewal marker Nanog, OCT4, ALDH1, and CD44 in a dose dependent manner. Data shown are representative of three individual experiments.

To determine whether STAT3 pathway activity is required for the survival or self-renew of tumor cells, we used S3I-201, a novel inhibitor of STAT3 by blocking dimerization [10] for *in vitro* functional experiment. We started to examine the expression of p-STAT3 in HNSCC cell lines FaDu, SCC4, SCC9, UMSCC23, CAL27, SCC15 and SCC25 as compared with normal oral squamous epithelia keratinocyte (OKC). As shown in Figure 2A, high level p-STAT3 expression was detected in all HNSCC cell lines with even stronger level in CAL27 and FaDu as compared with control. We also examined the protein level of four self-renewal transcription factors: SOX2, CD44, ALDH1 and OCT4 (Supplementary Figure S3H) and got similar result with p-STAT3, and there is no change of the STAT3 protein level. Therefore, we selected CAL27 and FaDu cell lines with high phosphorylation of STAT3 for the following *in vitro* functional assay. We analyzed the cell viability of CAL27 using CCK8 kit in indicated concentrations of S3I-201. As shown in Figure 2B, S3I-201 inhibited CAL27 cell growth with IC50 of 99.3 uM. We confirmed this inhibition of cell viability by on target effect as indicated by decrease of p-STAT3 with S3I-201 by immunofluorescence using confocal scope (Figure 2C). To further confirm whether the inhibition of cell growth by S3I-201 was through apoptotic cell death, we performed flow cytometry. As shown in Figure 2D, STAT3 blockade could significantly increase the Annxin V^+^PI^+^ and Annxin V^+^PI^−^ cell population in a dose dependent manner after 24 h S3I-201 treatment. This result was also confirmed in other indicated time point (Supplementary Figures S3A and S3B) and was repeatable in another HNSCC cell line FaDu (Supplementary Figures S3C and S3D). To verify the effect of S3I-201 on self-renewal ability, we found that HNSCC CAL27 cells formed tumor-spheres was directly proportional to the number of cells seeded. As shown in Figure 2E, STAT3 blockade with S3I-201 could significantly reduce the size and number of tumor spheres which indicating the self-renewal or initiation ability when compared with control (Figure 2E). To further confirm the effect of STAT3 blockage, we examined the self-renewal markers by Western blotting (Figure 2F). As expected, S3I-201 could decrease the protein level of Cyclin D1 and Bcl-2, which are known as a putative downstream target of STAT3. Additionally, inhibition of STAT3 may decrease self-renewal marker Nanog, OCT4, ALDH1 and CD44. This result was also validated in another HNSCC cell line FaDu (Supplementary Figures S3E–S3G). The above results suggest that STAT3 blockade could not only decrease non-CSC cancer cells but also reduce CSCs through self-renewal transcription factors, indicating STAT3 blockade can truly inhibit CSCs phenotype with small molecule inhibitor S3I-201.

### Blockage of p-STAT3 reduces CSCs phenotype in xenograft mouse model of HNSCC

**Figure 3:**
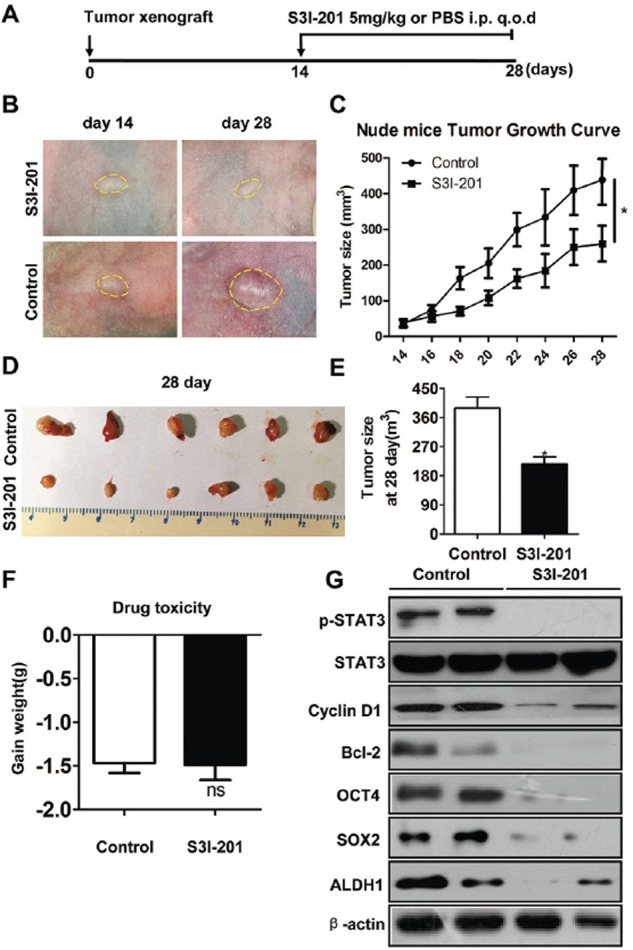
STAT3 inhibition reduces tumor growth and CSCs in HNSCC xenograft model **A.** Schematic diagram for the xenograft implantation and drug delivery. STAT3 signaling inhibitor S3I-201 (5 mg/kg) or equivalent volume PBS (control) was given by intraperitoneal injection (i.p) every other day (q.o.d) in CAL27 cells xenograft nude mice for consecutively 14 days (*n* = 6 mice, respectively). **B.** Representative images showed tumor regression in HNSCC xenograft treated with S3I-201 (upper) as compared with control group. Dahs lines were utilized to depict the outline of tumor lump. **C.** Total tumor volume was assessed in S3I-201 and control treatment every other day. *,*P* < 0.05. **D.** Representative images of tumor lump dissected from xenograft mice showed tumor harvest from nude mice with S3I-201 or control treatment. **E.** Tumor size from CAL27 xenograft in both S3I-201- and control-treated mice; *,*P* < 0.05 **F.** Drug toxicity was assessed by gained body weight of CA27 xenograft mice in each group. **G.** Western blotting shows S3I-201 decrease protein expression of p-STAT3, Cyclin D1, Bcl-2 and CSCs related markers OCT4, SOX2 and ALDH1 as compared with control group, while the protein level of STAT3 had no change. β-actin was used as loading control.

To evaluate the activity and tolerability of S3I-201 in HNSCC *in vivo*, we took advantage of human HNSCC CAL27 xenograft model. 10^6^ cells/mouse CAL27 cells were implanted subcutaneous into the flank of nude mice, and treatment was initiated beginning 14 days after tumor inoculation. As shown in schematic diagram Figure 3A, S3I-201 (5 mg/kg) or control (100 μl PBS) was given intraperitoneal (i.p.) every other day (q.o.d) for 14 consecutive days. Tumor volume and body weight were calculated every other day with electronic caliper and balance. As shown in Figure 3B and 3C, tumor volume had a remarkable reduction 14 days after S3I-201 treatment when compared with control group (Figure 3D and 3E). We evaluated the toxicity of this dosage with body weight changes. The result showed there was no significant additional toxicity of S3I-201 group as indicated by weight reduction as compared with control (Figure 3F, *P* > 0.05). To further confirm the decrease in tumor volume was directly correlated with CSCs blockade, tumor excised from xenograft mice were used to validated the correlation by Western blotting and immunohistochemistry. We compared the molecular expression of p-STAT3 in S3I-201 and control group. The data suggested that indeed S3I-201 could decrease p-STAT3 and its target gene Cyclin D1 and Bcl2 effectively *in vivo*, which indicate the decrease of tumor size of nude mice using S3I-201 is an on-target effect. CSCs marker ALDH1 and regulator OCT4, SOX2 were found reduced by Western blotting (Figure 3G) and by immunohistochemistry (Supplementary Figure S4A and quantification in Supplementary Figure S4B) after S3I-201 treatment. Taken together, these observations showed STAT3 blockade decreased tumor growth may through targeting apoptosis and CSCs phenotype in HNSCC xenograft mouse model.

### Blockade of p-STAT3 decreases CSCs phenotype in *de novo* spontaneous mice HNSCC

**Figure 4:**
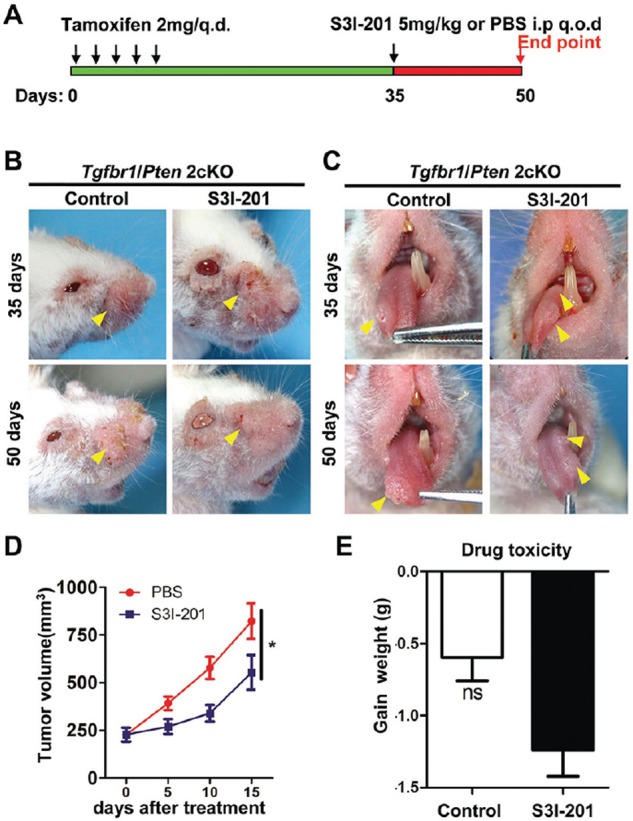
Chemotherapeutic treatment of S3I-201 in *Tgfbr1/Pten* 2cKO mice HNSCC **A.** Schematic diagram represent S3I-201 delivery strategy in *Tgfbr1/Pten* 2cKO mice. Oral application of tamoxifen was conducted consecutively 5 days. Mice receive 5 mg/kg S3I-201 or control PBS 100 μl intraperitoneal injection (i.p) every other day (q.o.d) for consecutively 15 days. Data present as mean ± SEM, *n* = 6, respectively. **B.** Representative photos show head and neck tumorigenesis was delayed after S3I-201 treatment 15 days as compared with control group. **C.** Representative photos show tongue tumorigenesis was delayed after S3I-201 treatment 15 days as compared with control group. **D.** Tumor volume curve showed S3I-201 treatment delay the growth of head and neck tumor; *,*P* < 0.05. **E.** Drug toxicity as indicated by gain of weight of S3I-201 and control treated mice. Ns, no Statistics.

To further determine the potential role of STAT3 pathway in tumor initiation effect, we take advantage of spontaneous *de novo* HNSCC mice models for our tumorigenesis studies. Combined epithelial knock out of *Tgfbr1* and *Pten* mice will full penetrated develop head neck and oral squamous cell carcinoma in a short period window (3–6 weeks after tamoxifen induction) [21]. *Tgfbr1/Pten* 2cKO mice were baseline induced with tamoxifen 2 mg for five consequently days to delete *Tgfbr1* and *Pten*. 5 mg/kg S3I-201 and control (100 μl PBS) were given i.p. every other day (q.o.d.) for 15 consecutive days (Figure 4A) for a chemotherapeutic experiment. Remarkably, S3I-201 treatment (*n* = 6) significant reduced head neck tumor (Figure 4B) and tongue tumor (Figure 4C) formation 14 days after infusion as compared with control group (*n* = 6, Figure 4D) without additional toxicity (Figure 4E). To further confirm the reduction of tumor was correlated with cancer stem cells, we used immunohistochemical staining to detect the p-STAT3 as well as CSCs markers OCT4, SOX2 and ALDH1. Similar to prior experiments OCT4, SOX2 and ALDH1 were consistently decreased after S3I-201 treatment when compared with control group (Supplementary Figure S5A and quantification in Supplementary Figure S5B). In addition, to further investigate the delay of tumor volume, we induced our mice model by tamoxifen administration and 14 days later we initiated treated with 5 mg/kg S3I-201 and control (100 μl PBS) were given i.p. every other day (q.o.d.) for 28 consecutive days (Supplementary Figure S6A) for chemopreventive experiment. Tumor volume was calculated once a week. S3I-201 treatment (*n* = 6) significant delayed head neck tumor (Supplementary Figure S6B) formation 6 weeks after intraperitoneal injection as compared with control group (*n* = 6, Supplementary Figure S6C) without additional toxicity (data not shown). Overall, these findings revealed that STAT3 blockade could delay tumor initiation and progression in *de novo* spontaneous mice model through reducing cancer stem cells.

### STAT3 inhibition attenuate chemo-reagent enriched HNSCC CSC population *in vitro* and *in vivo*

**Figure 5:**
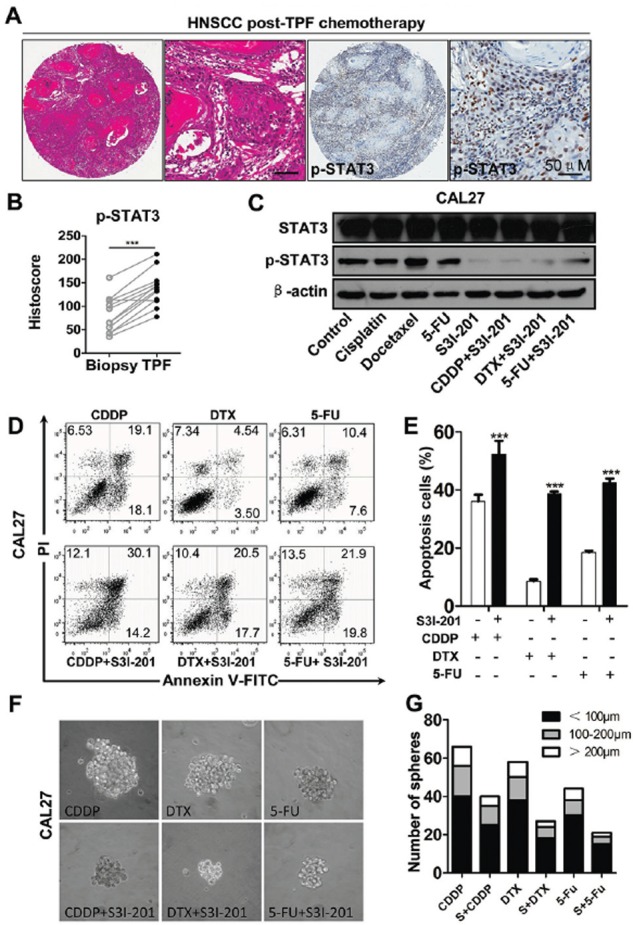
Increased expression of p-STAT3 in recurrence HNSCC after combined TPF chemotherapy and STAT3 signaling inhibition reduces chemoresistance of HNSCC *in vitro* **A.** Representative hematoxylin-eosin staining (HE) and IHC of p-STAT3^Tyr705^ significantly increased in HNSCC tissue post neoadjuvant TPF chemotherapy of cisplatin (CDDP), Docetaxel (DTX) and 5-fluoracil (5-FU) with quantification of histoscore in **B.** Data present as mean ± SEM, ***, *P* < 0.001. **C.** Single chemo reagents 10 μM cisplatin (CDDP), 10 μM Docetaxel (DTX) and 15 μM 5-fluoracil (5-FU) increase p-STAT3^Tyr705^ in CAL27 cell line, which may attenuate by combined S3I-201 (100 μM) treatment (24 h). **D.** Combinatorial conventional chemotherapy with S3I-201 increase apoptosis cell population of DTX, CDDP and 5-FU with quantification in **E. F.** *In vitro* tumor-sphere formation of DTX, CDDP and 5-FU with or without S3I-201 treatment. **G.** Quantification of tumor-sphere number of DTX, CDDP and 5-FU with or without S3I-201 treatment. Data shown are representative of three individual experiments.

**Figure 6:**
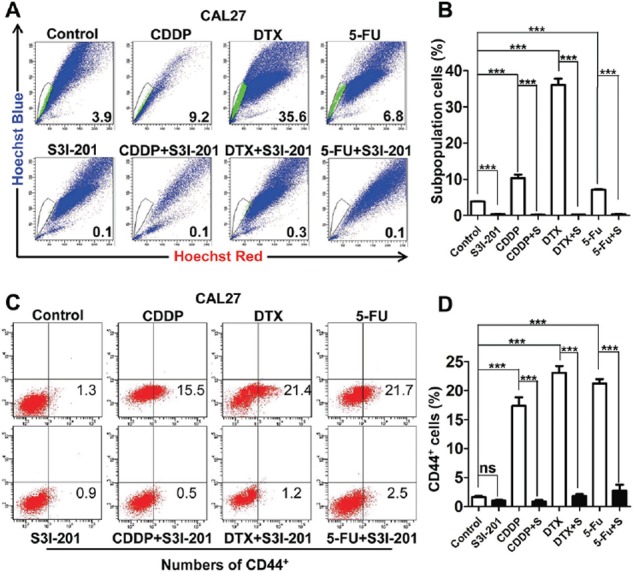
STAT3 inhibition attenuates chemo reagents enriched CSCs population *in vitro* **A.** Representative images of 10 μM cisplatin (CDDP), 10 μM Docetaxel (DTX) and 15 μM 5-fluoracil (5-FU) enrichment side population, which may attenuate by combined treatment of S3I-201. **B.** Quantification of side population. **C.** STAT3 inhibition by S3I-201 effectively reduced DTX, CDDP, 5-FU enriched CD44^+^ population as compared with single reagent counterpart. **D.** Quantification of side population. Data shown are representative of three individual experiments.

Myriad report suggested important function of chemo-resistant of CSCs and indicated chemotherapy enrich CSCs [22–24]. To determine the role of STAT3 pathway in chemotherapy, we utilized cherish paired specimen with induction combined TPF chemotherapy of CDDP, 5-FU, DTX of human HNSCC to validate our proposal. We found significant increased p-STAT3 expression level in post-TPF chemotherapy HNSCC as compared with biopsy specimen (Figures 5A and 5B). We analyzed the cell viability of CAL27 using CCK8 kit in indicated concentrations of TPF (Supplementary Figures S7A, S7B and S7C). We used CCK8 assay to analyze the cell viability after DTX treated by different concentrations of p-STAT3 inhibitor (Supplementary Figure S7D) and we confirmed this combination concentration of p-STAT3 inhibitor with TPF by Western blotting (Supplementary Figures S7E). Additionally, we found that phosphorylation of STAT3 was still high expressed in single chemo-reagent especially in DTX treated CAL27 cell line (Figure 5C), which suggest that activated STAT3 pathway may correlate with chemoresistance. To determine the combination effect of STAT3 inhibition with TPF regents, we used flow cytometry to detect the apoptosis of combined S3I-201 treatment. As expected combined S3I-201 treatment increased Annexin V^+^PI^+^ and Annexin V^+^PI^−^ CAL27 cell population (Figures 5D and 5E). To further confirm STAT3 inhibition may effect on tumor cell self-renewal ability, *in vitro* tumor-sphere formation assay was assessed for chemotherapeutical agents or in combination with S3I-201. As expected, S3I-201 combined with chemotherapeutical agents could decreased not only the size of tumor spheres but also the number of tumor spheres no matter the different size profile (Figures 5F and 5G). Consistent with our aforementioned experiments, S3I-201 combination with chemotherapeutical agents significantly decreased side population cells, which efflux the Hoechst dye via the ATP-binding cassette (ABC) family of transporter proteins expressed within the cell membrane, enriched by CDDP, DTX and 5–FU (Figures 6A and 6B). We then used CD44^+^ positive cells to further analyze the effects of STAT3 signaling in CSCs *in vitro* (Figures 6C and 6D). Collectively, combination of STAT3 inhibition may reduce classical TPF chemo-reagents enriched CSCs population *in vitro*.

**Figure 7:**
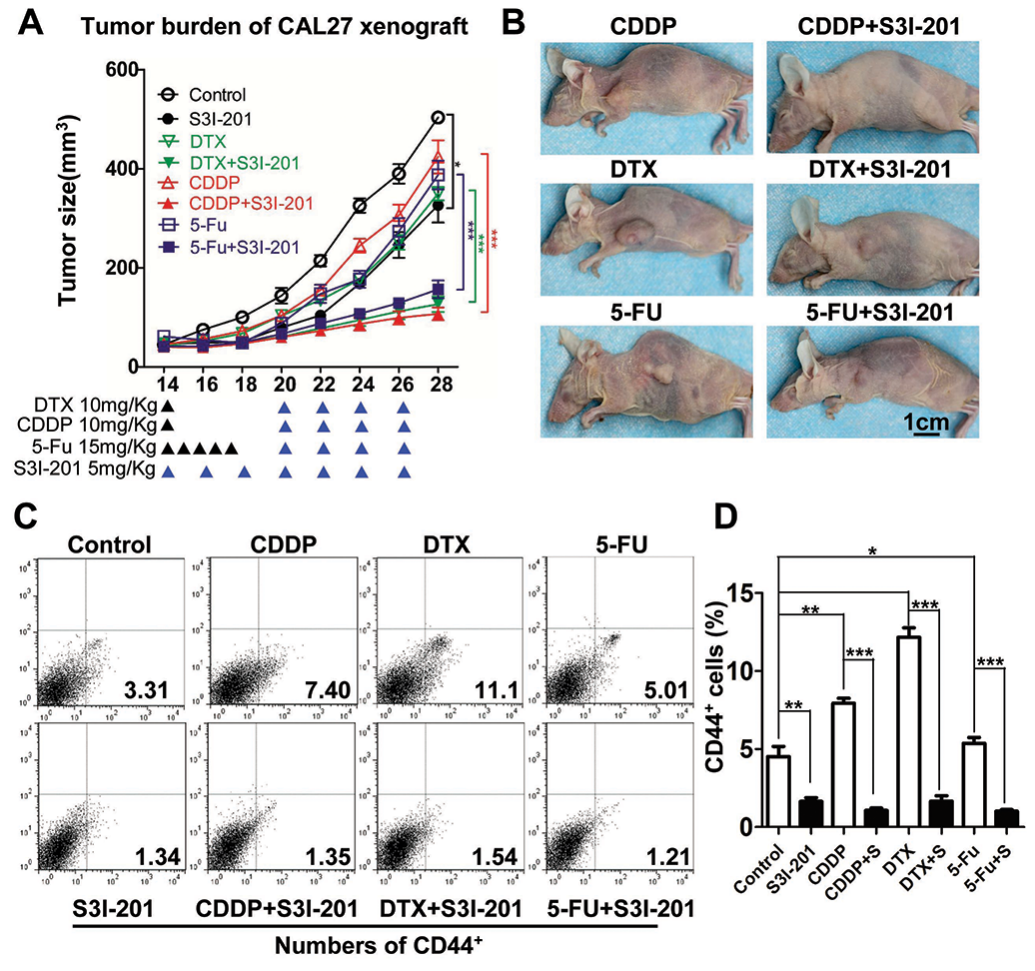
Combined STAT3 inhibition with TPF chemotherapy enhance anti-tumor effect *in vivo* **A.** Tumor growth curve and **B.** representative photo of DTX, CDDP, 5-FU, S3I-201 or combinational chemotherapy. 10 mg/kg DTX or CDDP were infused at day 14 and 15 mg/kg 5-FU was infused every day from day14 to day 19. The S3I-201 only group receive 5 mg/kg S3I-201 every other day and combined group receive additional 5 mg/kg S3I-201 every other day (as indicated by blue ▲) from day 21.*n* = 5 mice respectively. **C.** The xenografts from nude mice receiving DTX, CDDP, 5-FU, S3I-201 or combinational chemotherapy and analyzed by CD44^+^ flow cytometry. **D.** Quantification of CD44+ population. Data present as mean ± SEM, *, *P* < 0.05; **, *P* < 0.01; ***, *P* < 0.001.

To further determine whether the STAT3 inhibition reduces chemo-reagents enriched CSC *in vivo*, we used a xenograft mice model. We started single reagent chemotherapy of DTX, CDDP and 5-FU respectively with or without S3I-201 treatment 14 days after inoculation. A schematic *in vivo* drug delivery strategy was described in Figure 7A according to literature [25] and modified from inductive TPF chemotherapy protocol of human HNSCC patient [26]. For chemotherapeutic reagent treatment 10 mg/kg CDDP or DTX were infused at day 14, while 15 mg/kg 5-FU was infused every day from day14 to day 19. The S3I-201 only group tumor bearing mice receive 5 mg/kg S3I-201 every other day and combined group receive additional 5 mg/kg S3I-201 every other day from day 20 to day 26 (*n* = 5 mice respectively). As shown in Figure 7A and 7B, the S3I-201 only group are quite consistent with previous experiment with slightly significant (*P* < 0.05) reduction of tumor volume as compared with control (PBS) group. While, combinatorial chemotherapy with S3I-201 may act synergistically to reduce tumor growth as compared with single reagent groups (*P* < 0.001 in CDDP, DTX and 5-FU group respectively). To confirm the reductions of tumor growth is on-target effect of CSCs, we harvested the xenograft from mice and analyzed with flow cytometry. As shown in Figure 7C, combined S3I-201 treatment significantly reduced TPF-regents enriched CD44^+^ cell population. Quantification of CD44^+^ was shown in Figure 7D. Briefly, we confirm combinational STAT3 inhibition by S3I-201 may significantly reduce tumor growth by reducing HNSCC CSCs population *in vitro* and *in vivo*.

## DISCUSSION

CSCs or cancer stem-like cells has been thought to be responsible for the relapse and metastasis of cancer [27] and has been regarded as a novel target for cancer therapy [8]. On the basis of the increased expression of p-STAT3 in human HNSCC, which is consistent with previously study, we hypothesized that STAT3 may play an important role in maintaining HNSCC cancer stem cell properties. Indeed, we found that p-STAT3 expression is critical for maintaining stemloid cancer cell properties [27], including tumor initiation and resistance to chemotherapeutics in HNSCC. Furthermore, we observed STAT3 blockade delayed the *de novo* tumorigenesis of mice HNSCC by reducing of stemloid cancer cell. The role of STAT3 in HNSCC is also highlighted by increased tumoricidal effect of STAT3 inhibitor and reduction of enriched CSCs in combined with CDDP, DTX and 5-FU chemotherapy.

As a point of convergence for many oncogenic signaling pathways, STAT3 plays an important roles in angiogenesis, immune regulation and maintains stemloid cancer cell by crosstalk with driver mutation events of HNSCC e.g. *Notch1*, *P53*, *NF-κB* and *EGFR* (see review [28]). Indeed, The identification of CSCs and their roles has altered our understanding of cancer biology and caused a reevaluation of current therapies in HNSCC [3]. Although it's rather clear that STAT3 signaling play a crucial role in tumor initiation [29, 30], viability, angiogenesis and metastasis [28, 30–32], the function of STAT3 signaling in HNSCC CSCs is still an open question. Consistent with Grandis and colleagues [31], the present study also demonstrated that blockade of STAT3 can significantly induced cell death in HNSCC cell lines and obviously decreased tumor volumes in xenograft mice model. More interestingly, reduced tumor growth by STAT3 signaling blockade was coupled with remarkable inhibition of cancer stem cells implicated by tumor sphere and CSCs self-renewal markers. Indeed, the *de novo* spontaneous HNSCC mice chemopreventive study showed more significant reduction of tumor growth when compared with the late stage chemotherapeutic experiment, which indicated early inhibition of both CSCs and bulk tumor cells with p-STAT3 inhibitor S3I-201 may be responsible for the increased effectiveness. This significant inhibition of tumorigenesis in early stage of STAT3 inhibition may be due to significant activation of mTOR in the early stage of tumorigenesis of *Tgfbr1/Pten* 2cKO mice [21] and positive feed forward loop of mTOR/STAT3 [33]. Also, it indicated that the debulking effect of STAT3 inhibition in mice HNSCC model was rather a comprehensive effect on CSCs, proliferation, angiogenesis [34] and tumor infiltrating immune cells [28]. Recent reports indicated that angiogenic genes are important characteristic molecular signature of HNSCC CSCs [35] and immune cells [36] such as immature myeloid cells which play an important role in maintaining cancer cell stemness. All above, our results revealed that blockade of STAT3 could target both non-CSCs to decrease tumor growth and CSCs to generate powerful treatment in the early stage of tumor.

Moreover, the existence of CSCs is the central contributing factor to hinder successful cancer chemotherapy [37]. We speculated that targeting STAT3 pathway may be an effective therapeutic strategy for HNSCC with chemotherapeutic enriched CSC properties. Recent study report that, the proportion of the CD44^+^/CD24^−^ CSCs were significantly increased in the specimens treated with neoadjuvant chemotherapy compared with paired breast cancer biopsy specimens without chemotherapy [38]. Consistent with this report, the current study demonstrated the expression level of p-STAT3 in post-TPF chemotherapy was significantly higher than original HNSCC. Indeed, cytotoxic reagent CDDP not only enrich HNSCC CSCs by debulking proliferating cancer cell but also increase the fraction of stem cell by inducing putative self-renewal marker Bmi1 [39] and associated with STAT3 activation [40]. The most common sign of multidrug-resistance is the increased drug efflux from cancer cells by ATP-binding cassette (ABC) transporters, which was highly expressed in CSCs and was used to identify and isolate side population cells [41, 42]. In our study, we found TPF chemotherapy could significantly enriched the SP cells, which were eliminated by the combination of p-STAT3 inhibitor S3I-201 with TPF *in vitro*. We demonstrated that S3I-201 could significantly enhance the conventional chemotherapeutic agents by eradicating CSCs. Recent report indicate STAT3 play a pivotal role in IL6/STAT3/Jagged-1/Notch feedback loop in chemoresistance [43]. As mentioned above, STAT3 signaling has been linked to chemoresistance of CSCs, suggesting that inhibition of STAT3 signaling may not only disrupt the maintenance of CSCs [44] but also reduce the chemoresistance of CSCs triggered by CDDP, DTX, and 5-FU.

Knowledge of STAT3 signaling pathway allows numerous pharmaceutical development strategies to suppress STAT3 activation such as (a) inhibiting the receptor ligand complexes; (b) blocking the kinases that phosphorylate the cytoplasmic tail of the receptor; (c) inducing the activity of the phosphatases that dephosphorylate STAT3; (d) inhibiting JAK kinases thereby stopping STAT3 dimerization; (e) preventing nuclear translocation of STAT3; (f) blocking STAT3 DNA binding and transcriptional activity; (g) application of STAT3 anti-sense strategies and (h) decoy oligodeoxynucleotides [45]. Although, transcription factors such as STAT3 have traditionally been deemed “undruggable”, there are small molecule tyrosine kinase inhibitors that have been designed to inhibit the STAT3 pathway including AG490 and AZD1480, and several of these agents are being studied in clinical trial setting [46]. STAT3 decoy oligonucleotide binds specifically to deactivate STAT3 and blocks binding of STAT3 to DNA sequences on STAT3-responsive promoters, resulting in inhibition of STAT3-mediated transcriptions [47], but unlucky, this preclinical trials utilizing STAT3 inhibitors had to date been disappointing. S3I-201 is a new discover small molecule of inhibiting STAT3 dimerization and thereby prevent nuclear translocation of STAT3 [10], and so far there is no clinical trial on this molecule. In our study, S3I-201 could significantly reduce tumor cells *in vitro* and attenuate tumor burden *in vivo* without additional side effect through an on target effect by decreasing the level of p-STAT3. It is well known that p-STAT3 is required in early development and STAT3-null mice suffer embryonic-lethal and non-tumor cells can survive *in vitro* and *in vivo* by specific knocking out stat3 [48]. In our *in vivo* study, S3I-201 didn't reduce tumor-bearing mice body weight. However, as a ubiquitous transcription factor, more relatively experiments deserved to do to verify its potential toxicity. Recent advancements in nanomedicine [49] might be a potential strategy in reducing possible toxicities by allowing tumor-specific delivery of S3I-201 and even reduced the drug dose of S3I-201.

Collectively, the current study, we showed that S3I-201 delayed tumorigenesis of mice HNSCC and enhanced the efficacy of conventional chemotherapeutic agents by eradicating CSCs in HNSCC. While, keep in mind that the promising preclinical data was not guarantee to have good efficacy in human as STAT3 decoy treatment in mice [47] and human [17] HNSCC. Our study suggests that treatment with STAT3 signaling antagonists alone or in combination with the conventional chemotherapy agents may offer improved treatment for HNSCC and warrant future clinical trial.

## MATERIALS AND METHODS

Detailed materials and methods see supplementary files.

### Spontaneous HNSCC mouse models

All experiments were conducted in accordance with guidelines of the Institutional Animal Care and Use Committee of the Wuhan University. The inducible tissue-specific *Tgfbr1/Pten* 2cKO mice (*K14-Cre*^ERtam+/−^; *Tgfbr1*^flox/flox^; *Pten*^flox/flox^) were maintained and genotyped according to published protocols [21, 50]. All animal studies were carried out in compliance with the NIH guidelines for the use of laboratory animals in specific pathogen free (SPF) Animal Laboratory of Wuhan University School & Hospital of Stomatology as approved by the Animal Care and Use Committee of Wuhan University. The details of *Tgfbr1* cKO HNSCC mice (*K14-Cre*^ERtam+/−^; *Tgfbr1*^flox/flox^), *Pten* cKO HNSCC mice (K14-Cre^ERtam+/−^; *Pten*^flox/flox^) were previously described [21, 50]. All the mice were maintained in FVBN/CD1/129/C57 mixed background.

### S3I-201 treatment

S3I-201 (NSC74859) was purchased from Selleck Chemicals (Westlake Village, CA) and dissolved in dimethyl sulfoxide for use at indicated concentrations. For nude mice xenograft chemotherapeutic experiment, a flask of Human HNSCC cell line CAL27 were injected subcutaneously in nude mice. Two weeks after injection, the tumor was visible. The mice were randomly divides into control group (PBS, i.p. q.o.d, *n* = 6 mice) and 5 mg/kg S3I-201 treated group (i.p. daily, *n* = 6 mice) were performed with 14 days observation. For combined S3I-201 and chemotherapy reagent experiment, 10 mg/kg CDDP or DTX were infused at day 14, while 15 mg/kg 5-FU was infused every day from day14 to day 19. The S3I-201 only group tumor bearing mice receive 5 mg/kg S3I-201 every other day and combined group receive additional 5 mg/kg S3I-201 every other day from day 20 to day 26 (*n* = 5 mice respectively).

For chemotherapeutic transgenic mouse HNSCC tumorigenesis experiment, 4- weeks after the last dose of oral gavage of tamoxifen for 5 consequent days, the *Tgfbr1/Pten* 2cKO mice were randomly divided into experiment group receive 5 mg/kg S3I-201 intraperitoneal injection every other day (i.p. q.o.d, *n* = 6 mice) or control group (PBS, i.p. q.o.d, *n* = 6 mice). Mice were treated with this dosing schedule of S3I-201 for 25 days, and tumor size was measured every 5 days. For chemopreventive transgenic mice HNSCC tumorigenesis experiment, 1- week after the last dose of gavage tamoxifen, the *Tgfbr1/Pten* 2cKO mice were randomly divided into experiment group receive 5 mg/kg S3I-201 intraperitoneal injection every other day (i.p. q.o.d, *n* = 6 mice) or control group (PBS, i.p. q.o.d, *n* = 6 mice). S3I-201 and PBS treatment were performed at day 14 and maintain for 4 weeks. For all animal experiment tumor size was measured with a micrometer caliper and photographed every other day. The endpoint was determined according to a systematic evaluation by the veterinary doctor. The mice were euthanized using CO_2_ at the end of the studies, and the tumors were fixed in paraffin overnight or frozen at −80°C for the following immunohistochemical analysis.

### Cell culture, cell proliferation assay and Annexin V/PI staining

HNSCC cell lines CAL27, FaDu, SCC4, SCC9, SCC15, SCC25, UM-SCC23 were purchased from the American Type Culture Collection (ATCC, Manassas, VA). 2–3 passive primary cultured oral keratinocyte cell line (OKC) was used for a normal control. Cell proliferation was accessed by Cell Counting Kit (CCK8, Dojindo Laboratories, Japan) assay. Annexin V/PI (BD Pharmingen. San Diego, CA) staining was performed according to manufacturer's instruction and cell counted by flow cytometry (BD Pharmingen. San Diego, CA) [51].

### Sphere formation assay and side population assay

Tumor sphere culture assay were carried out as previously described [51]. Side population discrimination was based on Goodell and colleagues [52] with slight attention according to the recommendation of Boesch and colleagues [53]. Briefly, cells (1 × 10^6^ cells/mL) were resuspended in pre-warmed DMEM (Life Technologies) with 2% FBS (Life Technologies) containing freshly added Hoechst 33342 (5 μg/mL final concentration) for 90 minutes at 37°C in water bath with intermittent mixing and darkness, either alone or in the presence of 50 μmol/L verapamil (Sigma). At the end of incubation, samples were chilled on ice, centrifuged down at 4°C and resuspended in ice-cold PBS with 2% FBS (Life Technologies). 7-AAD at a final concentration of 2 μg/mL was added for 5 minutes before fluorescence-activated cell sorting (FACS) analysis, which allows for the discrimination of dead versus live cells. To dissociate multicellular aggregates, the cells were filtered with 40-μm cell strainer. The Hoechst33342 dye was excited with the UV laser at 355 nm and its fluorescence was dual-wavelength analyzed (blue, 450/65 nm; red, 670/30 nm) with FACSVantage SE (Becton Dickinson).

### Flow cytometry and western blot

Flow cytometry assay and Western blot were carried out as previously described [51].

### Histology, immunochemistry and immunofluorescence

Histology, immunochemistry and immunofluorescence were performed as previously described [51].

### Human HNSCC tissue microarray

The custom made HNSCC tissue microarrays of humans used in this study were described previously [21], with the approval of the Medical Ethics Committee of School and Hospital of Stomatology, Wuhan University. These tissue microarray slides included 43 confirmed cases of HNSCC, 16 normal oral mucosa and 6 oral epithelial dysplasia, 12 patients with biopsy and post-TPF chemotherapy specimen. The 12 HNSCC patients receive 2 round combined CDDP, DTX, and 5-FU therapy with the same protocol of Zhang's clinical trial [26]. Patient samples with both biopsy as well as surgical specimen after 2 rounds were involved in these custom-made tissue microarrays.

### Scoring system, hierarchical clustering, data visualization and statistical analysis

As we previously described [54], the hiotoscore of each slice was calculated as a percentage of different positive cells using the formula (3+) × 3+(2+) × 2+(1+) × 1, cluster program with average linkage based on Pearson's correlation, and data visualization were done using the Tree View program. Statistical data analysis was performed with GraphPad Prism 5.03 (GraphPad Software, Inc., La Jolla, CA). The differences in immunostaining and protein levels among each group were analyzed by the One-way ANOVA followed by the post-Tukey or Bonferroni multiple comparison tests. The Mann–Whitney U test was used to evaluate differences in the total tumor area of the mice treated with S3I-201 and control group. Two-tailed Pearson Statistics were used for correlated expression of these markers after confirmation of the sample with Gaussian distribution. Statistical significance was defined as the *p*-value was < 0.05.

## SUPPLEMENTARY MATERIALS AND METHODS, FIGURES


