# Supplementary material for: STAT3 blockade enhances the efficacy of conventional chemotherapeutic agents by eradicating head neck stemloid cancer cell
Source: Oncotarget. 2015 Nov 9;6(39):41944–58. doi: 10.18632/oncotarget.5986 (PMC4747200; doi:10.18632/oncotarget.5986)
Supplement: Supplementary file 1 [file oncotarget-06-41944-s001.pdf]

SUPPLEMENTARY MATERIALS AND METHODS

Cell culture, cell proliferation assay and Annexin V/PI staining

HNSCC cell lines CAL27, FaDu, SCC4, SCC9, SCC15, SCC25, UM-SCC23 were purchased from the American Type Culture Collection (ATCC, Manassas, VA). oral keratinocyte cell line (OKC) was used for a normal control. Cell lines were maintained in Dulbecco's modified Eagle's medium (DMEM)/F12, 10% fetal bovine serum (FBS), at 5% CO2 and 37°C humidified incubator with anti-vibration equipment. Cell proliferation was accessed by Cell Counting Kit (CCK8, Dojindo Laboratories, Japan) assay [1]. Briefly, samples of 5×10^3 cells/well for CAL27 were plated into 96-well plates in triplicate and were allowed to adhere overnight. After treated with indicated concentrations of S3I-201 in DMEM for 24 h. Media was removed and cells were resuspended with DMEM and 10% CCK8. After 2 h incubation, the absorbance value of each well was measured at 450 nm (excitation). Annexin V/PI (BD Pharmingen. San Diego, CA) staining was performed according to manufacturer's instruction and cell counted by flow cytometry (BD Pharmingen. San Diego, CA) [2].

Sphere formation assay

Tumor sphere culture assay were carried out as previously described [2]. Briefly, single-cell suspensions were resuspended in culture media containing 2% B27 supplement (Gibco), 20 ng/mL basic fibroblast growth factor (bFGF, R&D), and 20 ng/mL epithelial growth factor (EGF, R&D) and plated in ultra-low attachment plates (Corning) at a density of 5×10^3 cells per well. For subsequent passaging the spheres were collected and dissociated and then sieved through a 40-µm strainer. Dissociated cells were washed with serum-free Hank's balance salt solution for cultured in ultra-low attachment plates for subsequent experiments [3]. Medium was replenished twice a week and spheres counted within 2 weeks. The number and size of spheres was evaluated using an inverted microscope.

Western blot

The Western blot analysis was performed according to our previous procedures [4]. Briefly, about 30ug of protein from each sample were denatured and then loaded in each lane of 10% Bis-Tris precast gel. Subsequently, proteins were transferred onto a membrane and blocked for 1 hour, then incubated with primary antibodies overnight, finally by incubation with horseradish peroxidase-conjugated secondary antibody (Pierce, Rockford, IL). Next, the blots were using an enhanced chemiluminescence detection kit (West Pico, Thermo). The following primary antibody dilutions were used: 1:1000 for STAT3, p-STAT3^{Tyr705}, Cyclin D1, Bcl2, Nanog, OCT4, SOX2, ALDH1. β-actin was used as a loading control, which detected on the same membrane.

Cell immunofluorescence and confocal microscopy

In order to immunofluorescence staining [2], CAL27 cells were seeded on Coverglass Bottom Dish (Confocal Dish, BD-Falcon). After designed treatment, the cells fixed by 4% paraformaldehyde at room temperature for 15 min, and then treated with 0.1% triton X-100. After blocked with 2.5% BSA for 1 h, the cells were incubated with primary antibody overnight at 4°C. The cells were incubated with secondary antibodies with 4′,6′-diamidino-2-phenylidole (DAPI) for 1 h. The slides were observed by a confocal microcopy (Olympus, Tokyo, Japan). Representative cells were selected and provided.

REFERENCES

1. Liu N, Zhong L, Zeng J, Zhang X, Yang Q, Liao D, Wang Y, Chen G, Wang Y. Upregulation of microRNA-200a associates with tumor proliferation, CSCs phenotype and chemosensitivity in ovarian cancer. Neoplasma. 2015.
2. Ma SR, Wang WM, Huang CF, Zhang WF, Sun ZJ. Anterior gradient protein 2 expression in high grade head and neck squamous cell carcinoma correlated with cancer stem cell and epithelial mesenchymal transition. Oncotarget. 2015; 6:8807–8821.
3. Sun S, Liu S, Duan SZ, Zhang L, Zhou H, Hu Y, Zhou X, Shi C, Zhou R, Zhang Z. Targeting the c-Met/FZD8 signaling axis eliminates patient-derived cancer stem-like cells in head and neck squamous carcinomas. Cancer Res. 2014; 74:7546–7559.
4. Sun ZJ, Zhang L, Hall B, Bian Y, Gutkind JS, Kulkarni AB. Chemopreventive and chemotherapeutic actions of mTOR inhibitor in genetically defined head and neck squamous cell carcinoma mouse model. Clin Cancer Res. 2012; 18:5304–5313.

SUPPLEMENTARY FIGURES

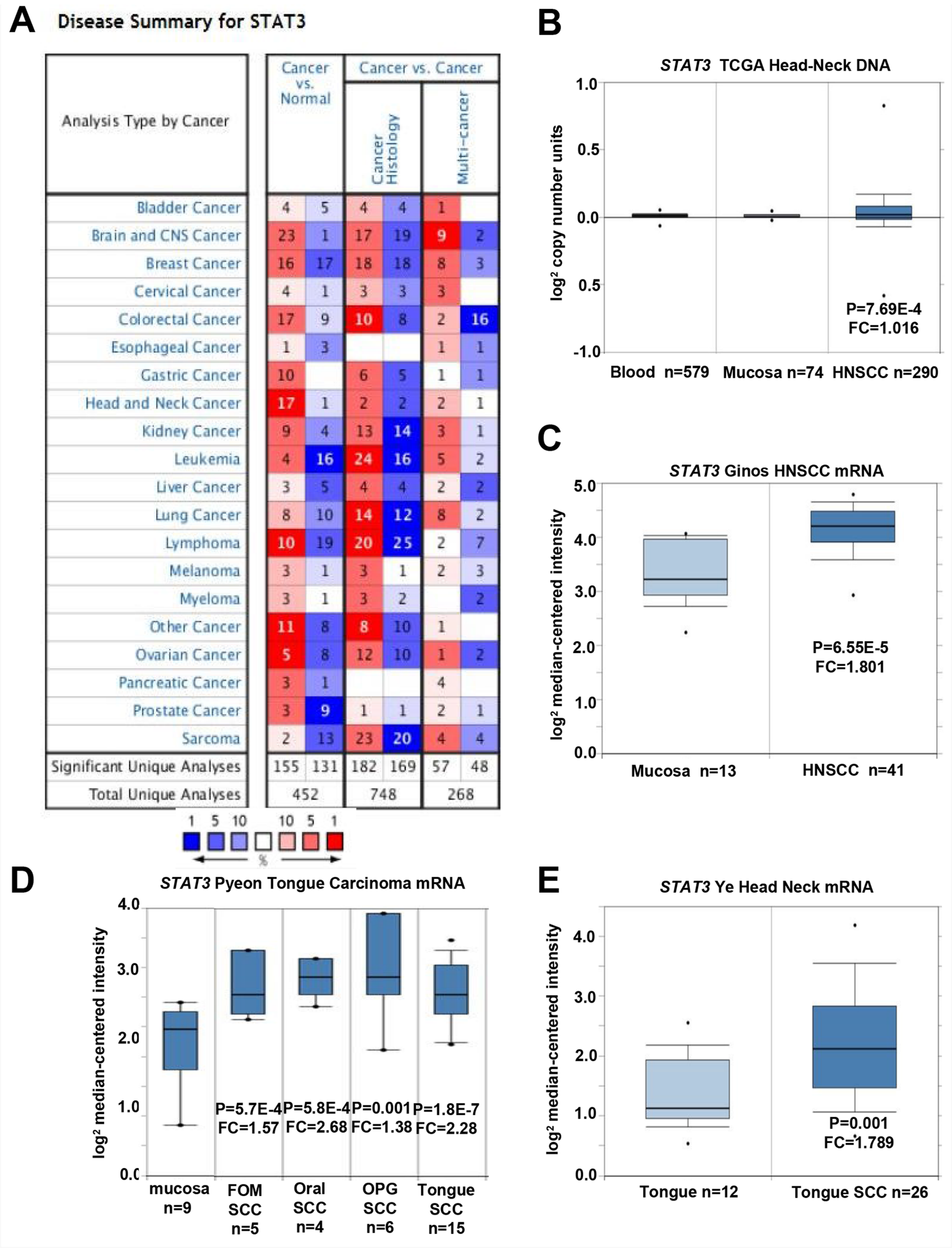

Supplementary Figure S1: Increased *STAT3* DNA copy number and mRNA level in head neck cancer. A. Disease summary of STAT3 retrieve from Oncomine database indicate high expression of STAT3 is an important molecule event in head neck cancer. **B.** DNA copy number of *STAT3* in TCGA dataset shows increase *STAT3* copy number of HNSCC as compared with mucosa and blood. mRNA level of *STAT3* in Ginos's dataset **C.** Pyeon's dataset **D.** and Ye's dataset **E.** shows as normalized data of log2 median–centered ratio.

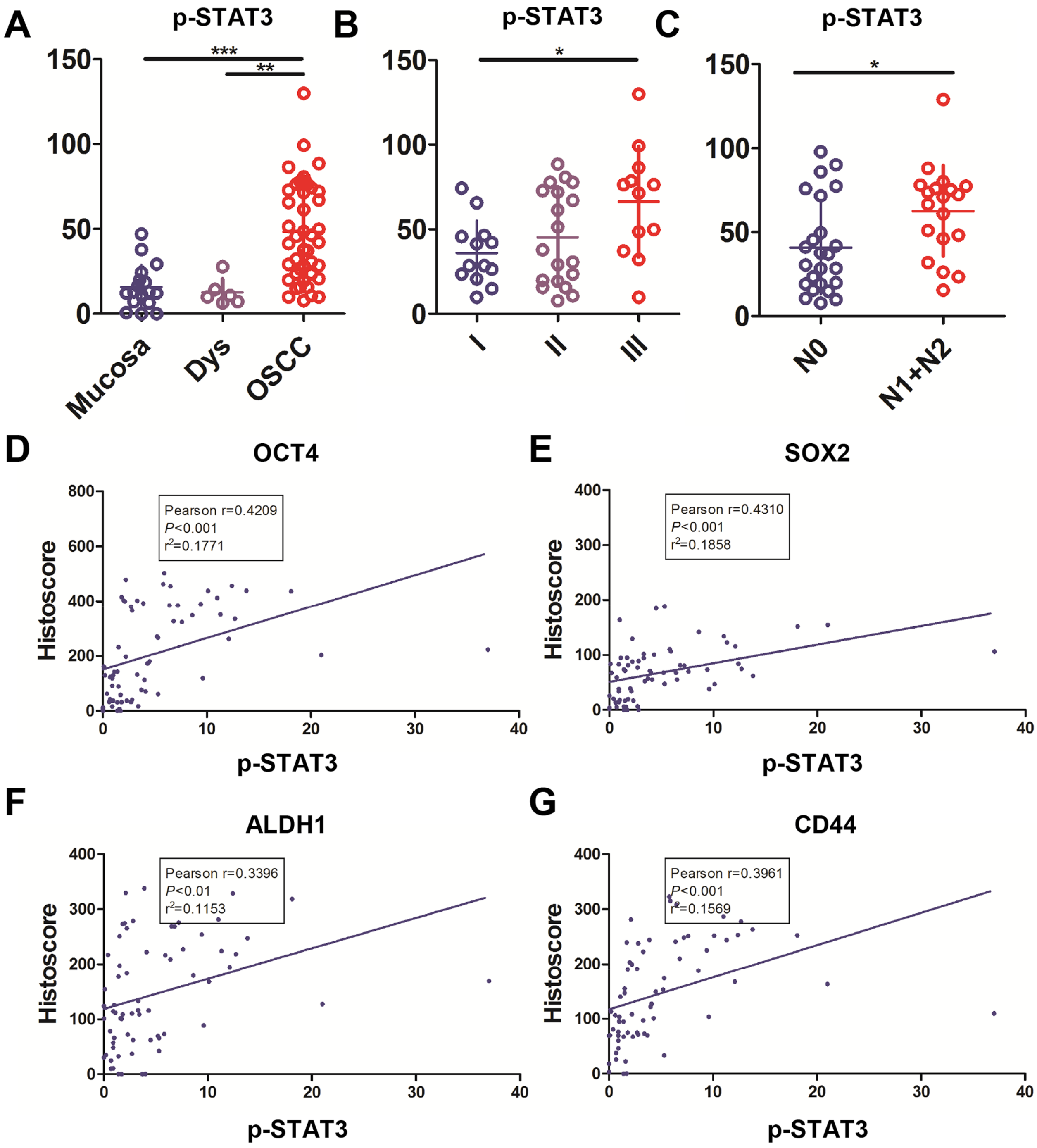

Supplementary Figure S2: Activated STAT3 signaling correlates with CSCs markers in HNSCC. As shown in Figure **A.** Over-expression of p-STAT3^{Tyr705} in oral squamous cell carcinoma as compared with dysplasia (Dys, n = 6) and normal oral mucosa (Mucosa, n = 16); Data present as mean ± SEM, **, $P < 0.01$; ***, $P < 0.001$. **B.** Over-expression of p-STAT3^{Tyr705} correlated with high grade OSCC; *, $P < 0.05$, while there is no significant difference between Grade III and Grade II, and no significant difference between Grade > and Grade >. **C.** Over-expression of p-STAT3^{Tyr705} in original OSCC correlated with draining lymph node Status; *, $P < 0.05$. Phosphorylation of STAT3 expression was correlated with OCT4 (**D.** $r = 0.4209$, $P < 0.001$), SOX2 (**E.** $r = 0.4310$, $P < 0.001$), ALDH1 (**F.** $r = 0.3396$, $P < 0.01$), CD44 (B, $r = 0.3961$, $P < 0.001$) in HNSCC determined by Pearson correlation analysis. Histoscore based on Aperio quantification and Statistics with GraphPad prism, two-tail Pearson correlation.

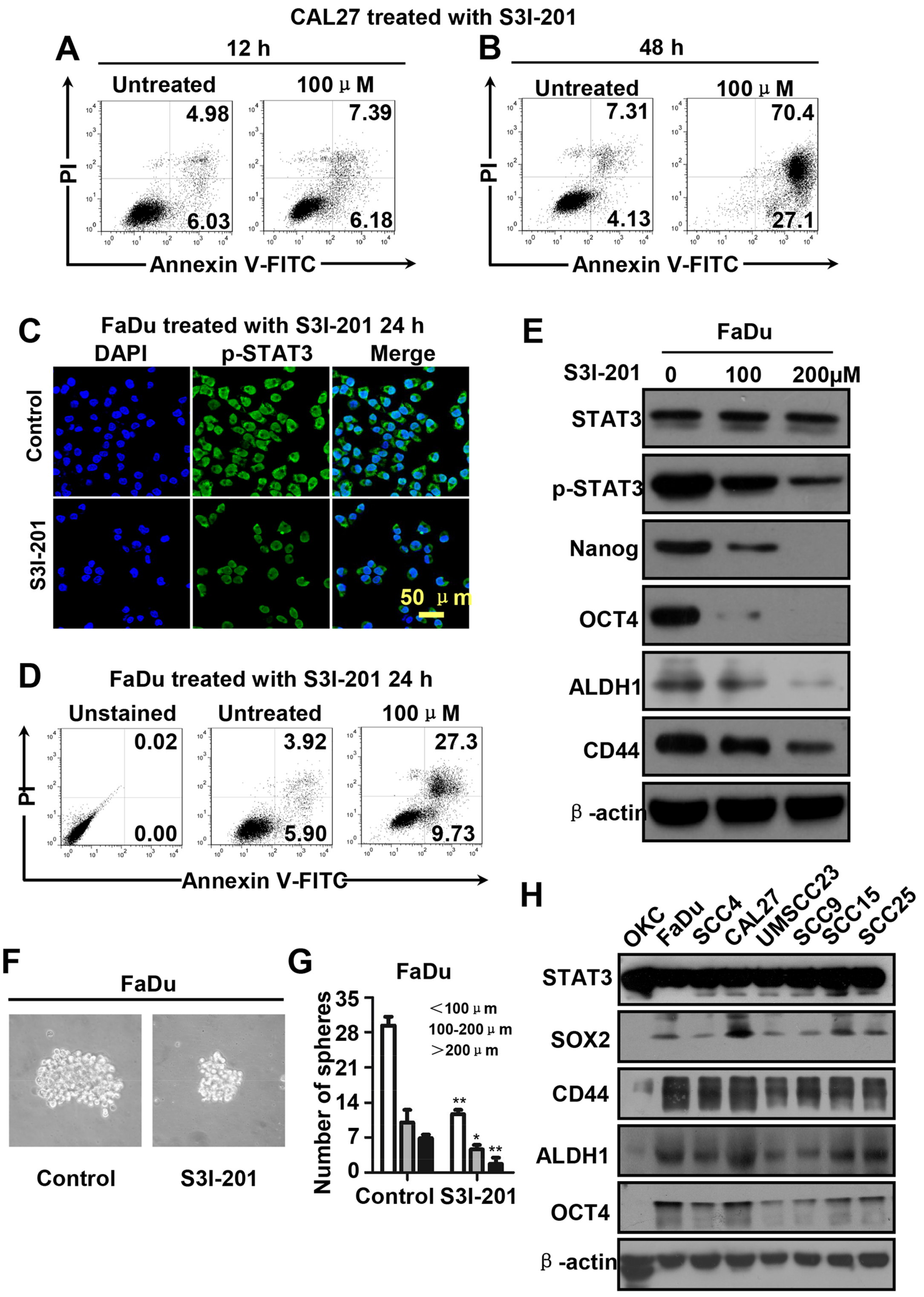

Supplementary Figure S3: STAT3 inhibition suppresses cell viability of HNSCC CAL27 and FaDu cell lines. Representative flow cytometry photo of S3I-201 treated CAL27 cell increase apoptosis cell population in 12 h **A.** and 48 h **B.** Data present as mean ± SEM, ***, $P < 0.001$. **C.** Representative immunoflurosence photo of S3I-201 treated FaDu cell for 24 h by confocal microscopy. **D.** Representative flow cytometry photo of S3I-201 treated FaDu cell increase apoptosis cell population in 24 h FaDu cell line. **E.** Western blot shows S3I-201 treatment decrease self-renewal marker Nanog, OCT4, ALDH1, and CD44 in a dose dependent manner in FaDu cell line. **F.** Sphere formation analysis shows S3I-201 decrease FaDu tumor-sphere formation. **G.** Quantification of tumor-sphere number *, $P < 0.05$; **, $P < 0.01$. **H.** Western blotting of STAT3, ALDH1, CD44, OCT4 and SOX2 of HNSCC cell lines as compared with oral keratinocyte cell line (OKC). Data shown are representative of three individual experiments.

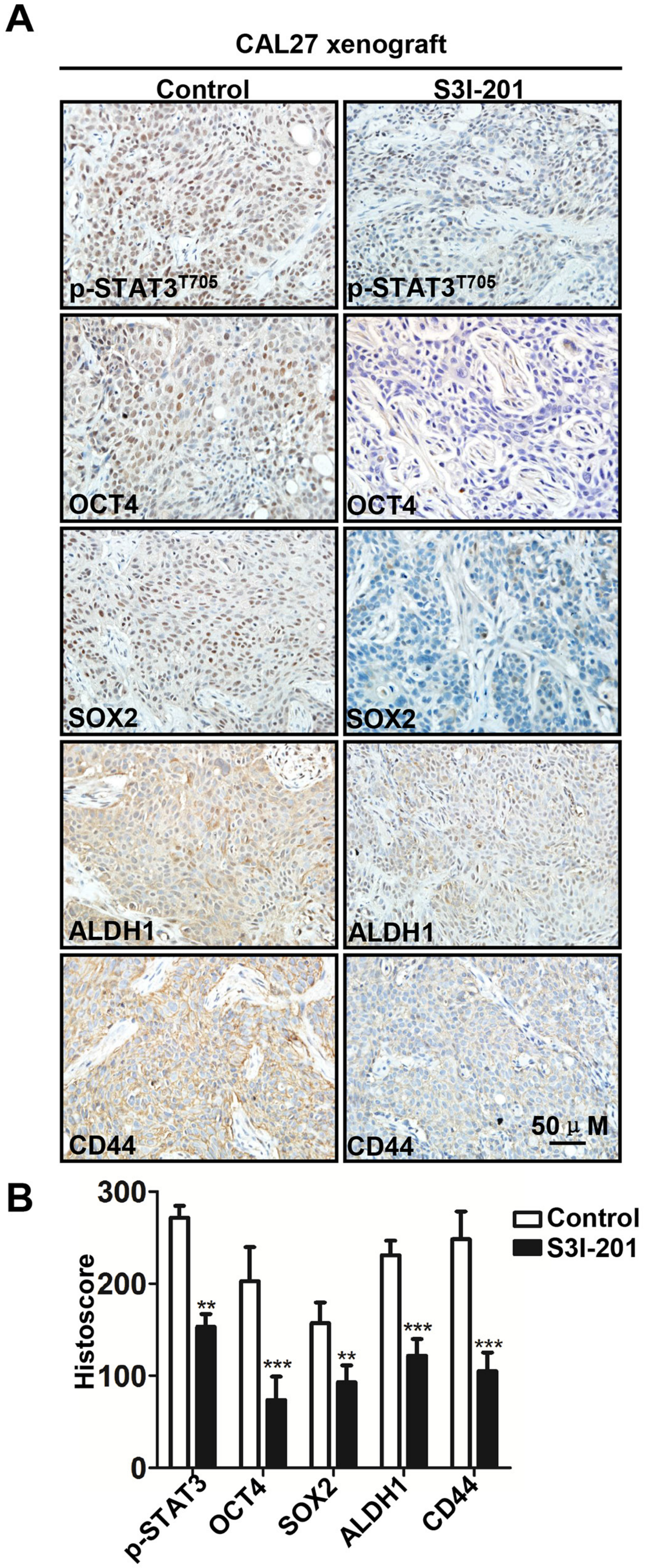

Supplementary Figure S4: STAT3 inhibition suppress CSC markers in CAL27 nude mice xenografts. A. reduced immunoreactivity of p-STAT3^{Tyr705}, OCT4, SOX2, ALDH1 and CD44 in S3I-201 treated xenograft (right) as compared with PBS only counterpart (left). Scale bar, 50 μm. **B.** Quantification of histoscore using Graph Pad Prism 5. Data present as mean ± SEM, **, *P* < 0.01; ***, *P* < 0.001

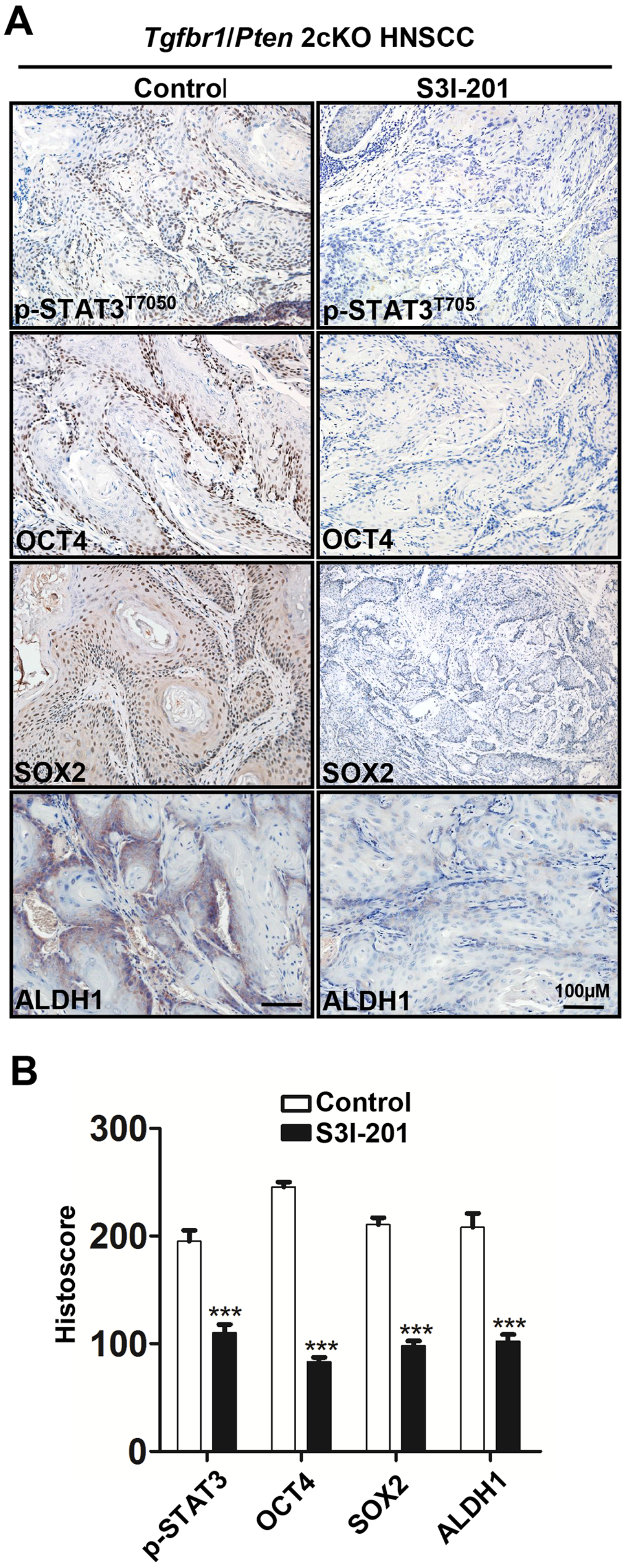

Supplementary Figure S5: STAT3 inhibition suppress CSC markers in spontaneous *de novo* mice HNSCC. A. reduced immunoreactivity of p-STAT3^{Tyr705}, OCT4, SOX2, ALDH1 in S3I-201 treated xenograft (right) as compared with PBS only counterpart (left) .Scale bar, 50 µm. **B.** Quantification of histoscore using Graph Pad Prism 5. Data present as mean ± SEM, ***, $P < 0.001$.

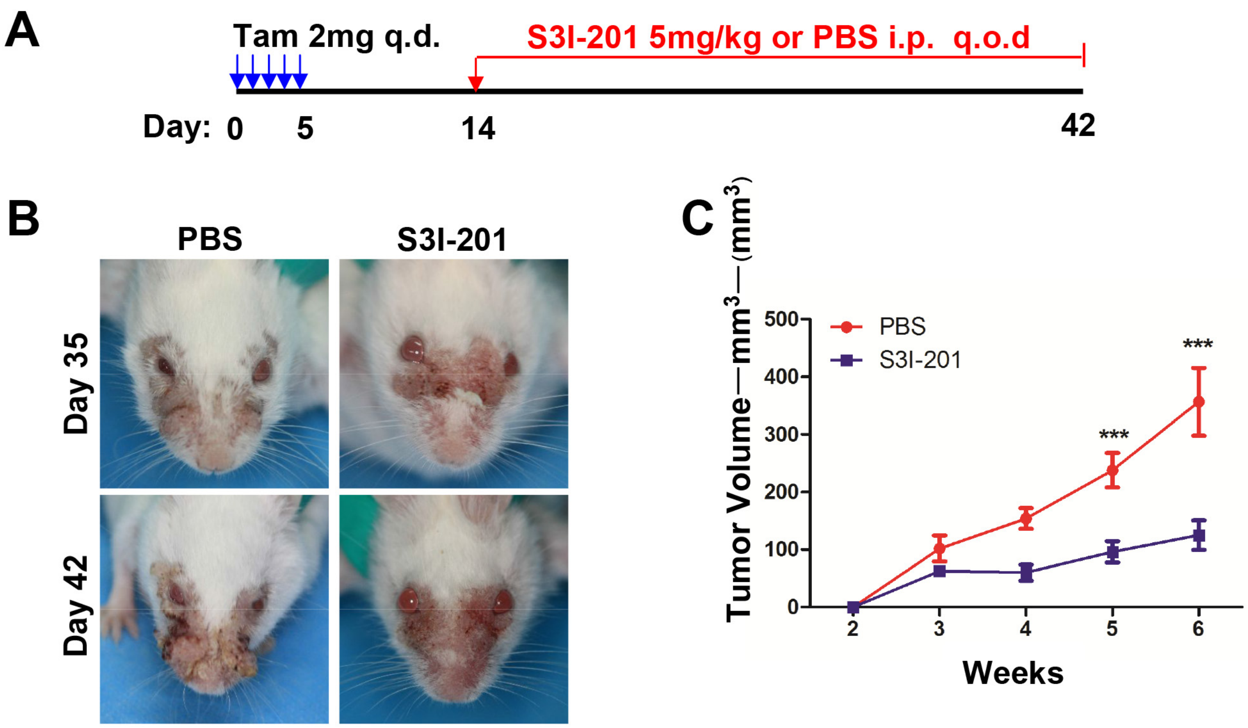

Supplementary Figure S6: Inhibition of p-STAT3 delays onset of *Tgfbr1/Pten* 2cKO mice HNSCC. A. A schematic showing a drug delivery strategy for S3I-201 in the chemopreventive tumorigenesis experiment in *Tgfbr1/Pten* 2cKO mice HNSCC. **B.** Head and neck tumorigenesis in chemopreventive tumorigenesis experiment. Upper panel shows representative photos of S3I-201 or control treatment 4weeks after tamoxifen induction, and lower panel shows photos 5 weeks after tamoxifen induction on the same mice. S3I-201 treatment significantly reduces head and neck tumor burden **C.** Data present as mean ± SEM, ***, $P < 0.001$.

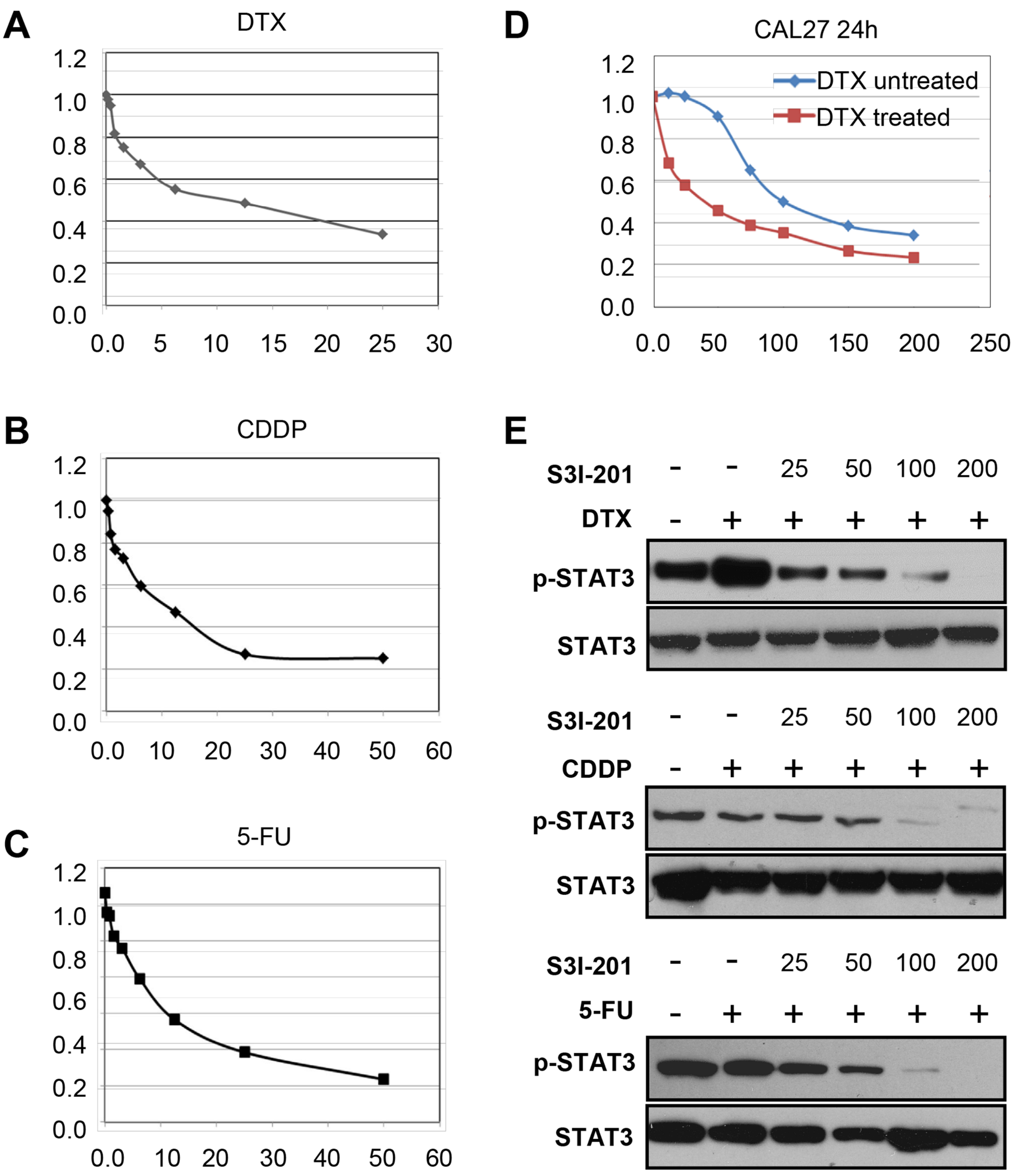

Supplementary Figure S7: Combination of p-STAT3 inhibitor and TPF in CAL27 cell line. Cell growth of CAL27 was measured using a CCK8 assay after DTX **A.** CDDP **B.** 5-FU **C.** treated for 24 h in different concentrations. **D.** Cell growth of CAL27 treated with DTX for 24 h and untreated with DTX was measured using a CCK8 assay after S3I-201 for 24 h in different concentrations. **E.** Western blot shows the combination use of different concentration p-STAT3 inhibitor with DTX (upper), CDDP (middle), 5-FU (below) in CAL27 cell line. Data shown are representative of three individual experiments.
